# Downregulation of monocytic differentiation via modulation of CD147 by 3-hydroxy-3-methylglutaryl coenzyme A reductase inhibitors

**DOI:** 10.1371/journal.pone.0189701

**Published:** 2017-12-18

**Authors:** Manda V. Sasidhar, Sai Krishnaveni Chevooru, Oliver Eickelberg, Hans-Peter Hartung, Oliver Neuhaus

**Affiliations:** 1 Department of Neurology, Heinrich Heine University, Düsseldorf, Germany; 2 Apollo Hospitals Educational and Research Foundation, Hyderabad, India; 3 Department of Internal Medicine II, Molecular Biology and Medicine of the Lung, Justus Liebig University, Giessen, Germany; 4 Wockhardt Research Centre, Aurangabad, India; 5 Division of Pulmonary Sciences and Critical Care Medicine, Department of Medicine, University of Colorado, Aurora, CO, United States of America; 6 Department of Neurology, SRH Kliniken Landkreis Sigmaringen, Sigmaringen, Germany; Klinikum rechts der Isar der Technischen Universitat Munchen, GERMANY

## Abstract

CD147 is an activation induced glycoprotein that promotes the secretion and activation of matrix metalloproteinases (MMPs) and is upregulated during the differentiation of macrophages. Interestingly, some of the molecular functions of CD147 rely on its glycosylation status: the highly glycosylated forms of CD147 induce MMPs whereas the lowly glycosylated forms inhibit MMP activation. Statins are hydroxy-methylglutaryl coenzyme A reductase inhibitors that block the synthesis of mevalonate, thereby inhibiting all mevalonate-dependent pathways, including isoprenylation, *N*-glycosylation and cholesterol synthesis. In this study, we investigated the role of statins in the inhibition of macrophage differentiation and the associated process of MMP secretion through modulation of CD147. We observed that differentiation of the human monocytic cell line THP-1 to a macrophage phenotype led to upregulation of CD147 and CD14 and that this effect was inhibited by statins. At the molecular level, statins altered CD147 expression, structure and function by inhibiting isoprenylation and *N*-glycosylation. In addition, statins induced a shift of CD147 from its highly glycosylated form to its lowly glycosylated form. This shift in *N*-glycosylation status was accompanied by a decrease in the production and functional activity of MMP-2 and MMP-9. In conclusion, these findings describe a novel molecular mechanism of immune regulation by statins, making them interesting candidates for autoimmune disease therapy.

## Background

Monocytes and macrophages play important roles in the pathogenesis of inflammatory diseases such as multiple sclerosis (MS) and rheumatoid arthritis (RA) [1,2,3]. In MS, macrophage differentiation and activation have been demonstrated to induce demyelination [4], and in RA, the number of macrophages infiltrating into synovial tissue correlates with the extent of local disease activity [5]. Activated macrophages are important sources of matrix metalloproteinases (MMPs); because of this, macrophages are the primary effectors of tissue destruction, and they participate in the degradation of both normal and abnormal matrix [6]. In inflammatory diseases like MS and RA, therapeutic strategies aim to counteract macrophage homing, activation and differentiation [3]. Furthermore, macrophages are key players in the development and progression of atherosclerosis, meanwhile acknowledged as a chronic inflammatory disease [7].

CD147, also known as extracellular matrix metalloproteinase inducer (EMMPRIN), is a cell surface glycoprotein that is expressed on activated monocytes and macrophages [3,8]. It has been demonstrated to stimulate the production of several MMPs, including MMP-2 [9,10] and MMP-9 [11,12]. Both MMPs contribute to the pathogenesis of MS: MMP-2 participates in the remodeling of the extracellular matrix in chronic inflammatory processes, and MMP-9 predominates in acute MS lesions [13]. MMP-9 also participates in generating autoimmunity in MS by cleaving myelin compounds [14]. Imbalances in MMP activity have also been implicated in RA [15]. MMP-2 and MMP-9 contribute to joint destruction by directly degrading cartilage and bone and by promoting angiogenesis [15]. Several MMPs including MMP-2 and MMP-9 contribute to atherosclerosis by initiation of collagen breakdown in plaques [16].

CD147 undergoes extensive post-translational processing and exhibits remarkable variations in its size, which ranges from approximately 31 to 65 kDa [17]. It has a glycan content of 5–35 kDa [18]. The highly glycosylated, high molecular weight forms induce MMP production, whereas the lowly glycosylated, low molecular weight forms have been shown to inhibit MMP function [9,18,19,20].

**Fig 1:**
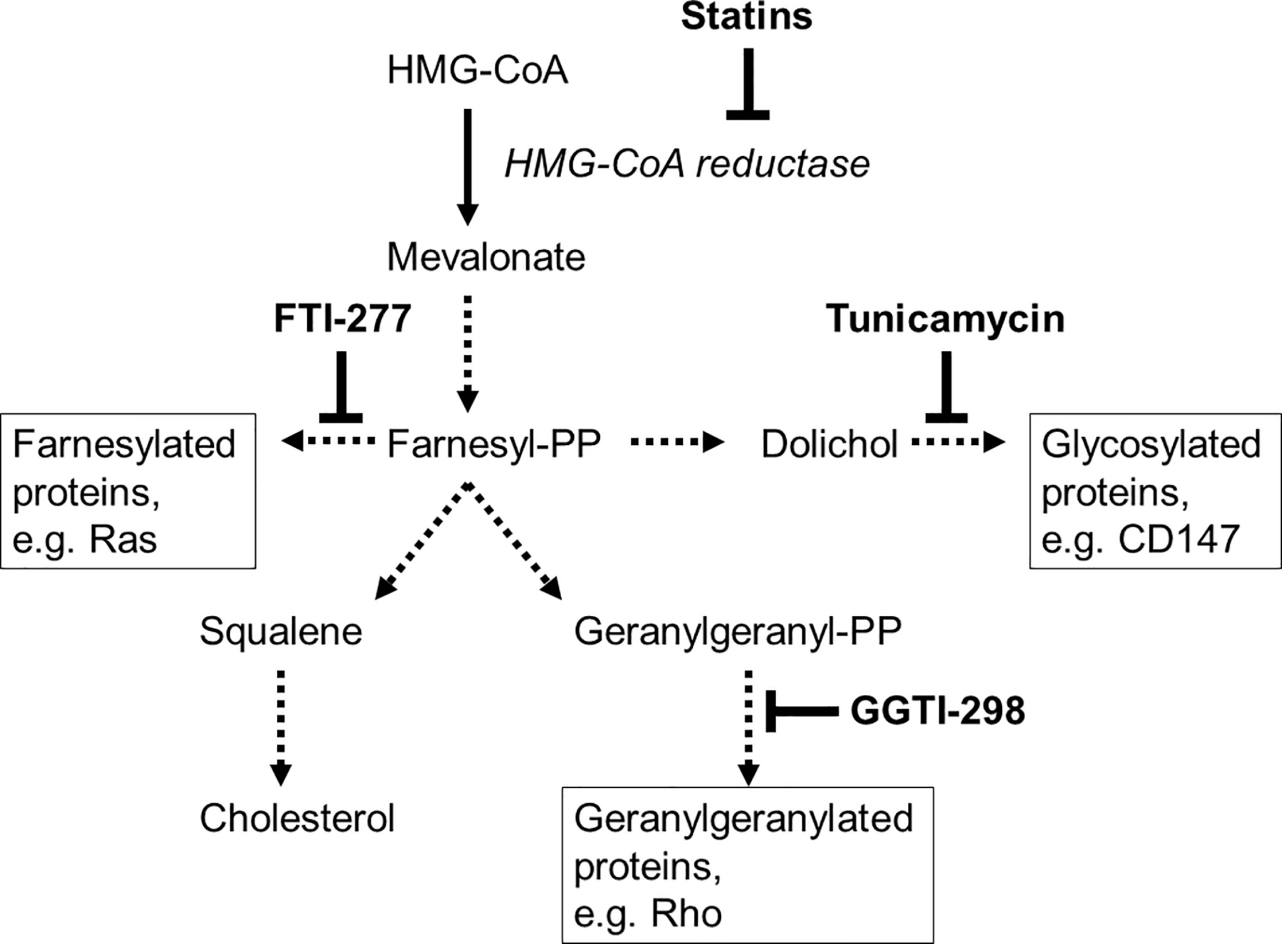
Statins and the cholesterol biosynthesis pathway. Statins inhibit 3-hydroxy-3-methylglutaryl coenzyme A (HMG-CoA) reductase-mediated formation of mevalonate, which is the rate-limiting step of the cholesterol biosynthesis pathway. Farnesylpyrophosphate (-PP) and its derivative, geranylgeranyl-PP, are lipids that posttranslationally modify immunologically important guanosine triphosphate (GTP)-binding proteins, such as Ras and Rho. This isoprenylation permits the subsequent activation and membrane translocation of these proteins, which is necessary for a number of their cellular functions. Dolichol production is dependent on the presence of farnesyl-PP, and dolichol is a necessary substrate for the *N*-glycosylation of immunologically important transmembrane glycoproteins such as CD147. Inhibitors of the production of specific intermediates downstream of mevalonate include the farnesyl transferase inhibitor (FTI)-277, the geranylgeranyl transferase inhibitor (GGTI)-298, and tunicamycin, a selective inhibitor of *N*-glycosylation.

Statins are inhibitors of 3-hydroxy-3-methylglutaryl coenzyme A (HMG-CoA) reductase, which catalyzes the rate limiting step in the cholesterol biosynthesis pathway [21]. Apart from their cholesterol lowering activity, statins have been demonstrated to have immunomodulatory effects *in vitro* and in animal models; if they prove effective in controlled trials, statins may serve as treatment options for MS or RA in the future [22,23,24,25,26,27,28]. For atherosclerosis prevention, statins are already widely used [29]. Statins block the formation of cholesterol biosynthesis pathway intermediates such as isoprenoids and dolichol (Fig 1). Isoprenoids derived from farnesylpyrophosphate (FPP) or geranylgeranylpyrophosphate (GGPP) form lipid attachments that are important for the membrane translocation of small GTP binding proteins [30]. Only upon isoprenylation are GTP binding proteins able to signal to their downstream effectors and promote vital cellular functions. Conversely, if isoprenylation is inhibited by statins, inactive forms of these GTP binding proteins accumulate in the cytosol, keeping the cell in a dormant state. Furthermore, reduction of FPP by statins results in the depletion of dolichol, which acts as a carbohydrate donor during the *N*-linked glycosylation of membrane targeted proteins. Lower levels of *N*-glycosylation result in the accumulation of immature forms of these proteins in the endoplasmic reticulum [31,32].

In this study, we investigated the role of statins in the inhibition of monocyte differentiation and the associated process of MMP secretion through modulation of CD147.

## Methods

### Reagents and antibodies

#### Reagents

Fluvastatin (Novartis, Nürnberg, Germany) and atorvastatin (Pfizer, Karlsruhe, Germany) were dissolved in dimethylsulfoxide (DMSO, which was used as a vehicle control; Sigma, St. Louis, MO) and used at concentrations ranging from 0.1 to 10 μM. Pravastatin (Merck, Darmstadt, Germany) was dissolved in water and used at the same concentrations. Mevalonate, squalene, and water-soluble cholesterol (Sigma) were used at a final concentration of 100 μM. Farnesylpyrophosphate (FPP), geranylgeranylpyrophosphate (GGPP; Sigma), and dolichol (Larodan, Malmö, Sweden) were all used at 10 μM. The following inhibitors of the cholesterol biosynthesis pathway were used at 10 μM: farnesyl transferase inhibitor (FTI)-277, geranylgeranyl transferase inhibitor (GGTI)-298, and the farnesyl/geranylgeranyl transferase inhibitor FPT-1 (all from Merck). Tunicamycin (Sigma), a potent inhibitor of the *N*-glycosylation of newly formed proteins [17], was used at a concentration of 10 μg/ml. AP-9 is a peptide antagonist of CD147 [33] (Biologisch-Medizinisches Forschungszentrum, Düsseldorf, Germany). Its purity, as assessed by high pressure liquid chromatography, was >95%.

#### Antibodies

An anti-human FITC-labeled anti-CD147 monoclonal antibody (clone MEM 6/1, mouse IgG1; Immunotools, Friesoythe, Germany) and an allophycyanin (APC)-labeled anti-CD14 monoclonal antibody (MɸP9, IgG2b; Becton Dickinson, San Jose, CA) were used for flow cytometry. The anti-CD147 monoclonal antibody (MEM 6/1, IgG1; Immunotools) was also used for immunoblotting.

### Cell culture

All experiments were performed in the human monocytic cell line THP-1 [8,11] (a kind gift from Dr Angelika Bierhaus, University of Heidelberg, Germany). Cells were cultured in RPMI 1640 medium supplemented with 5% FCS (Gibco BRL, Gaithersburg, MD), 1% penicillin/streptomycin and 2% L-glutamine (Gibco) at 37°C in a humidified atmosphere of 5% CO_2_. For induction of THP-1 differentiation, cells (2–3 x 10^6^) were seeded in the presence of 200 nM phorbol-12-myristate-13 acetate (PMA; Merck) and incubated for 24 h [11]. After incubation, non-attached cells were removed by aspiration, and the adherent cells were washed three times with medium. Undifferentiated THP-1 cells (seeded and incubated without PMA) were used as a control.

### Cytofluorometric analysis

The expression patterns of CD147 and CD14 were determined by flow cytometry. THP-1 cells (10^5^ per well) were washed with PBS (PAA Laboratories, Pasching, Austria) staining buffer containing 2% FCS and incubated with their respective antibodies for 45 min. After being washed three times with staining buffer, cells were analyzed in a FACScan^®^ instrument (Becton Dickinson) using FlowJo^®^ software (Treestar, Ashland, OR).

### Cell permeabilization assay

THP-1 cells were incubated with statins or controls and differentiated with PMA to upregulate CD147. Cells were simultaneously permeabilized and fixed in cell permeabilization buffer containing paraformaldehyde (BD Cytofix/Cytoperm^®^, Becton Dickinson). Flow cytometry was used to monitor the intracellular expression of CD147.

### CD147 immunoblotting

THP-1 cells were preincubated with statins or controls and differentiated with PMA for 24 h. Cells were then lysed in 1 ml RIPA buffer supplemented with sodium orthovandate (Sigma) and a complete protease inhibitor cocktail (Roche, Indianapolis, IN) for 45 min with intermittent shaking on ice. The proteins were separated on a 10% SDS-PAGE gel and transferred to a PVDF membrane using a Transblot instrument (Biorad, Hercules, CA) at 20 V for 1 h. The immunoblot was blocked for 2 h in 5% lipid free milk and incubated with an anti-CD147 antibody (Immunotools) for 1 h, followed by a 30 min wash. The primary antibody was conjugated to goat anti-mouse secondary antibody-horseradish peroxidase (HRP; Becton Dickinson) and incubated for 1 h. Finally, the blot was developed using an ECL development system (Amersham, Buckinghamshire, UK) or an enhanced ECL system (Pierce, Rockford, IL). Images were analyzed using ImageJ software (NIH, Bethesda, MD).

### Gelatin zymography

Cellular MMP production was measured in THP-1 cell supernatants. To assess MMP secretion, supernatants were collected after incubation with statins or controls, and MMP activity was determined by SDS-polyacrylamide gel zymography. Samples were centrifuged to remove cellular debris, and supernatants were collected and stored at -20°C. Ten μl of supernatant was mixed with 10 μl SDS loading buffer (Invitrogen, Carlsbad, CA) and loaded onto a 10% polyacrylamide gel containing 0.1% gelatin (Sigma). Positive controls for MMP-2 and -9 (R&D Systems, Minneapolis, MN) were added to each gel. After electrophoresis at 125 V for 150 min, the gel was renatured in a renaturating buffer (Invitrogen) containing 25% Triton X-100 for 30 min. After equilibration in developing buffer (Invitrogen) for 30 min, fresh developing buffer was added, and the gelatin containing gel was allowed to develop overnight at 37°C. The gelatin gels were stained with 0.5% Coomassie blue (Sigma) and destained in a buffer consisting of 10% acetic acid, 50% methanol and 40% distilled water for 30 min to visualize the zymogen bands produced by MMP digestion. An image of each gel was scanned after drying. Images were analyzed using ImageJ software (NIH).

### Membrane biotinylation

To assess the proportion of CD147 molecules that reached the cell surface of THP-1 cells, 5 x 10^6^ cells were seeded in a 40 ml dish, differentiated with PMA and treated with 10 μM of tunicamycin or the indicated statin. Cells were then washed three times with cold PBS and slowly agitated with 1 mg/ml biotin (Sulfo-NHS-Biotin; Pierce) for 30 min on ice. Cells were then lysed using lysis buffer containing 10% SDS, 0.4 M phenylmethylsulfonyl fluoride and 0.1 M benzamidine. Streptavidin agarose beads were washed in lysis buffer, mixed with the cell lysate and thoroughly mixed with the cell lysate. The biotinylated proteins were pulled down with beads and eluted in SDS Laemmli buffer for immunoblotting as described above.

### Statistics

Where applicable, Student’s T-test was performed for statistical analysis. A p-value of < 0.05 was accepted to be significant.

## Results

In this study, we examined the effect of statins on the structure and function of CD147 in the monocytic cell line model THP-1, which can be differentiated *in vitro* by treatment with PMA [11,34]. We used two approaches to further elucidate the pathways responsible for these effects: (i) rescue experiments (add-on approach) in which the processes inhibited by statins (i.e., isoprenylation and *N*-linked glycosylation involving mevalonate-derivative products) were selectively restored at key steps; and (ii) inhibition experiments, where the statin-induced effects were mimicked using known inhibitors of isoprenylation and *N*-linked glycosylation (Fig 1).

To this end, in all experimental setups, the following scenarios were investigated:

1. non-differentiated THP-1 cells were compared to differentiated cells;
2. the effects of statins on differentiated THP-1 cells were assessed;
3. rescue experiments were performed to reverse statin effects at different key steps of the cholesterol biosynthesis pathway;
4. the effects of statins were compared to those of inhibitors of processes downstream of HMG-CoA reductase.

### Statin treatment alters the morphology of PMA-differentiated THP-1 cells

**Fig 2:**
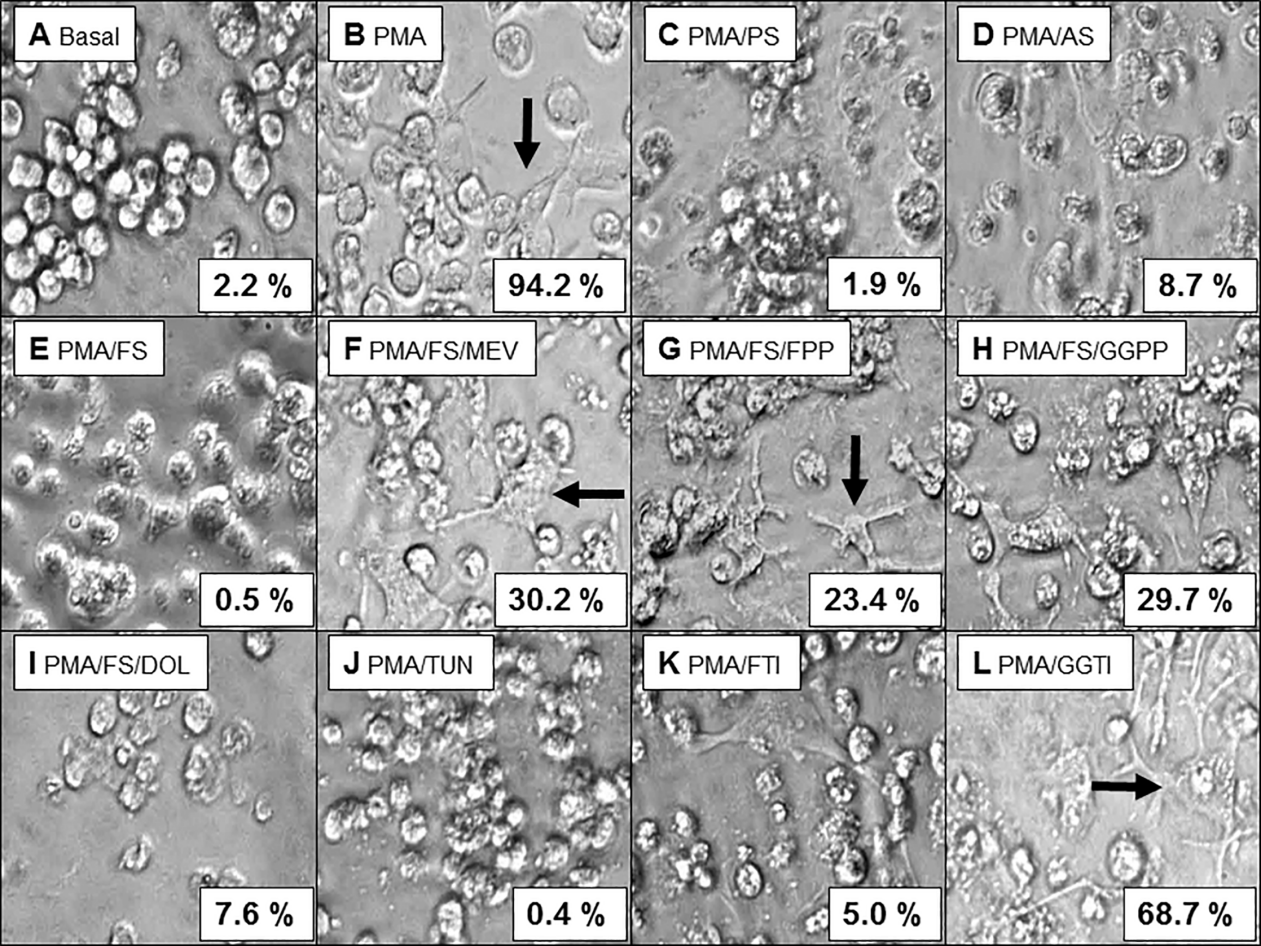
Alteration of the morphology of PMA-differentiated THP-1 cells by treatment with statins and various controls. THP-1 cells were (A) left untreated or (B-L) treated with 200 nM PMA for 24 h (B) without statins or in the presence of (C) 10 μM pravastatin (PS); (D) 10 μM atorvastatin (AS); (E) 10 μM fluvastatin (FS); (F) 10 μM fluvastatin plus 100 μM mevalonate (MEV); (G) 10 μM fluvastatin plus 10 μM farnesylpyrophosphate (FPP); (H) 10 μM fluvastatin plus 10 μM geranylgeranylpyrophosphate (GGPP); (I) 10 μM fluvastatin plus 10 μM dolichol (DOL); (J) 10 μg/ml tunicamycin (TUN); (K) 10 μM of the farnesyl transferase inhibitor (FTI)-277; or (L) 10 μM of the geranylgeranyl transferase inhibitor (GGTI)-298. Cellular morphology was assessed by light microscopy at 50x magnification. Numbers represent percentage of THP-1 cells with adherent, amoeboid morphology indicating differentiation (examples are marked by arrows). Experiments were conducted in triplicate; one representative result is shown.

In culture, naïve THP-1 cells are round and non-adherent (Fig 2A). Differentiation of THP-1 cells with PMA resulted in a change in morphology, with cells becoming flat, elongated, amoeboid and adherent (Fig 2B). In contrast, treatment with various statins (pravastatin, atorvastatin, and fluvastatin) followed by differentiation with PMA resulted in a cellular morphology similar to that of undifferentiated THP-1 cells; pravastatin treatment also resulted in the formation of bunched clusters of cells (Fig 2C–2E).

Next, rescue experiments were performed to reverse the effects of statins. Treatment with mevalonate, the product of HMG-CoA reductase, reversed the effect of fluvastatin and resulted in a differentiated cellular morphology (Fig 2F). Addition of downstream intermediates (FPP, GGPP, and dolichol) also reversed the effects of fluvastatin (although to decreasing degrees with dolichol exhibiting the weakest effects) and induced a differentiated cellular morphology (Fig 2G–2I).

Tunicamycin, an inhibitor of *N*-glycosylation, prevented differentiation of THP-1 cells in a similar manner to statins (Fig 2J). FTI-277 and GGTI-298, which inhibit isoprenylation, were partially able to prevent differentiation of THP-1 cells (Fig 2K and 2L).

### Statin treatment inhibits expression of CD147 and CD14 on PMA-differentiated THP-1 cells

**Fig 3:**
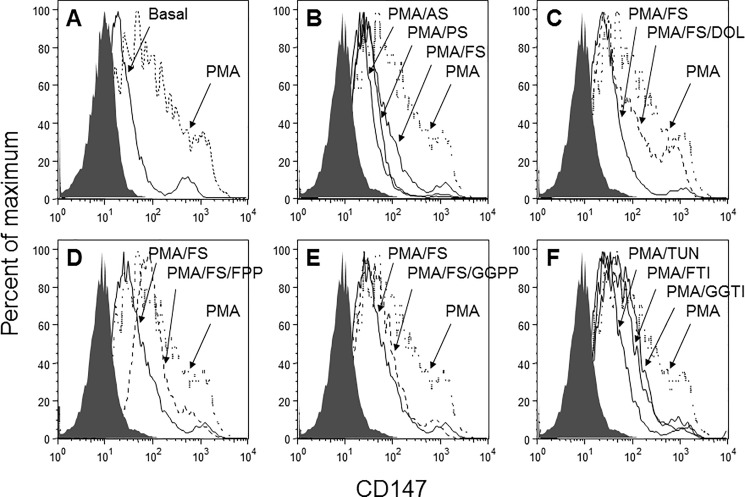
Alterations in CD147 expression on PMA-differentiated THP-1 cells upon statin and control treatments. The cell surface expression of CD147 on THP-1 cells treated with statins, rescue compounds, and cholesterol pathway inhibitors was analyzed by flow cytometry. THP-1 cells were left untreated or treated with 200 nM PMA for 24 h in the presence or absence of statins or controls. Changes in CD147 expression are shown in overlaid histograms. A: PMA-differentiated cells versus untreated cells (basal). B: PMA-differentiated cells versus cells treated with PMA in the presence of atorvastatin (AS), pravastatin (PS), or fluvastatin (FS). C-D: Rescue experiments: C: PMA-differentiated cells versus cells treated with fluvastatin (FS) and fluvastatin-treated cells rescued with dolichol (DOL). D: PMA-differentiated cells versus cells treated with fluvastatin (FS) and fluvastatin-treated cells rescued with farnesylpyrophosphate (FPP). E: PMA-differentiated cells versus cells treated with fluvastatin (FS) and fluvastatin-treated cells rescued with geranylgeranylpyrophosphate (GGPP). F: PMA-differentiated cells versus cells treated with the cholesterol pathway inhibitors FTI-277, GGTI-298 or tunicamycin (TUN). Gray histograms represent isotype controls. Experiments were conducted in triplicate; one representative result is shown.

Flow cytometric analysis revealed that the surface expression of CD147 was upregulated in PMA-differentiated THP-1 cells (Fig 3A). Treatment of cells with pravastatin, atorvastatin or fluvastatin resulted in CD147 downregulation (Fig 3B). Rescue experiments revealed that treatment with dolichol (Fig 3C) or FPP (Fig 3D) rescued the expression of CD147, whereas GGPP treatment only partially rescued CD147 expression (Fig 3E). In the inhibitor experiments, tunicamycin treatment induced the most potent inhibition of CD147 expression, followed by FTI-277 and GGTI-298 (Fig 3F), indicating that farnesylation may be the dominant pathway regulating the expression of CD147.

**Fig 4:**
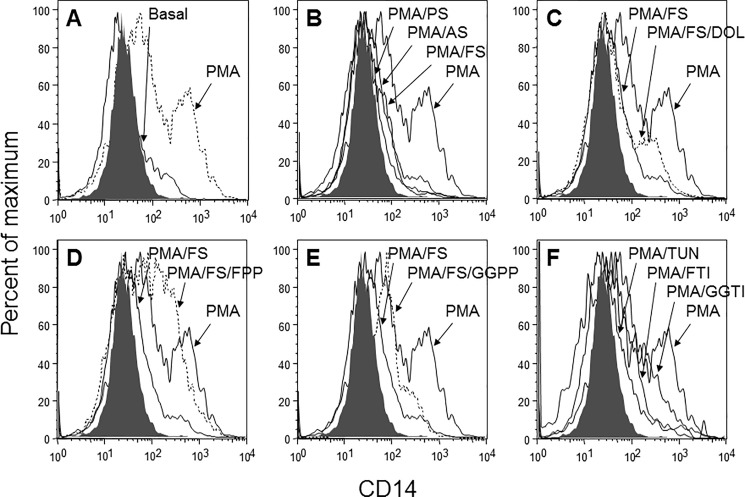
Alterations in CD14 expression in PMA-differentiated THP-1 cells induced by statin and control treatment. The cell surface expression of CD14 in THP-1 cells treated with statins, rescue compounds, and cholesterol pathway inhibitors was analyzed by flow cytometry. THP-1 cells were left untreated or treated with PMA in the presence or absence of statins or controls. The changes in CD14 expression are shown in overlaid histograms. A: PMA-differentiated cells versus untreated cells (basal). B: PMA-differentiated cells versus cells treated with PMA in the presence of atorvastatin (AS), pravastatin (PS), and fluvastatin (FS). C-E: PMA-differentiated cells versus cells treated with FS: FS-treated cells rescued with (C) dolichol (DOL), (D) farnesylpyrophosphate (FPP), or (E) geranylgeranylpyrophosphate (GGPP). F: PMA-differentiated cells versus cells treated with the cholesterol pathway inhibitors FTI-277, GGTI-298 or tunicamycin (TUN). Gray histograms, isotype controls. Experiments were conducted in triplicate; one representative result is shown.

Expression of the monocytic differentiation antigen CD14 is regulated in a manner similar to that of CD147; we therefore monitored the expression of CD14 along with CD147. PMA-mediated differentiation was accompanied by increased CD14 expression (Fig 4A) that was inhibited by treatment with any of the three statins (Fig 4B). Rescue experiments confirmed that dolichol (Fig 4C) and FPP (Fig 4D) almost completely rescued the differentiation of THP-1 cells, whereas GGPP failed to do so (Fig 4E). In the inhibition experiments, the inhibitors tested had similar effects on CD14 and CD147 expression (Fig 4F).

**Fig 5:**
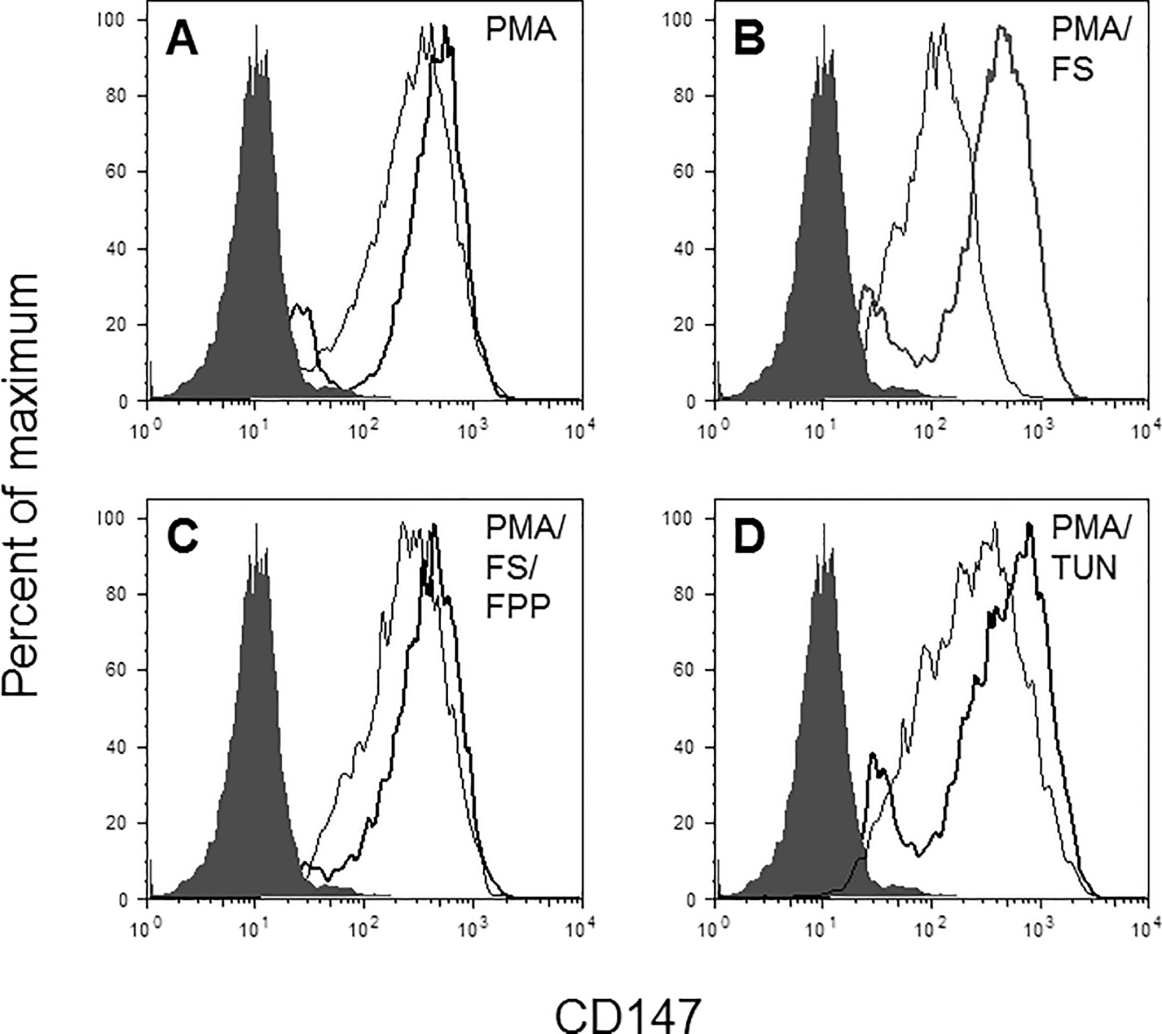
Effects of statins and controls on surface versus total CD147 expression. Cell permeabilization studies were conducted to confirm cellular retention of CD147. Results are shown as overlaid histograms. Light lines: surface-expression of CD147 in unpermeabilized THP-1 cells; bold lines: total cellular expression of CD147 in permeabilized cells. A: PMA-differentiated cells. B: PMA-differentiated cells treated with fluvastatin (FS). C: fluvastatin-treated, PMA-differentiated cells rescued with farnesylpyrophosphate (FPP). D: PMA-differentiated cells treated with tunicamycin (TUN). Gray histograms, isotype controls. Experiments were conducted in triplicate; one representative result is shown.

To assess if the downregulation of CD147 cell surface expression is caused by a decrease in overall protein levels or in membrane translocation, we next measured the expression of CD147 in permeabilized cells (which represents the total cellular level of CD147) and compared it to CD147 cell surface expression in non-permeabilized cells. These results demonstrate that in PMA-differentiated THP-1 cells, most of the CD147 molecules were expressed on the cell surface (Fig 5A), but that fluvastatin induced intracellular retention of CD147 (Fig 5B) that was reversed upon FPP treatment (Fig 5C). This effect of fluvastatin was mimicked by tunicamycin treatment (Fig 5D).

### Statins induce lowly glycosylated forms of CD147

**Fig 6:**
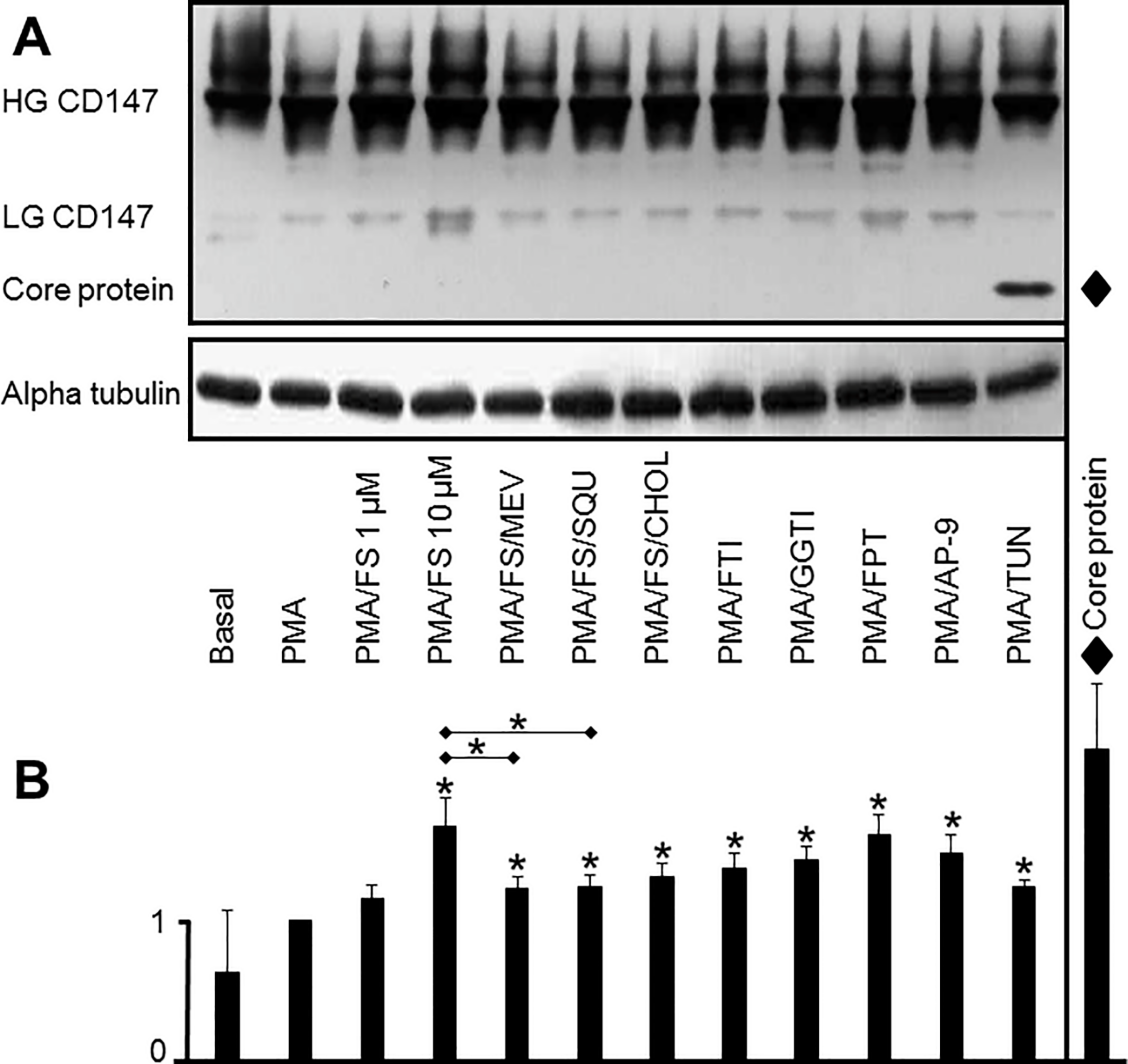
Effects of statins and controls on the glycosylation status of CD147. Total cell lysates were subjected to immunoblotting to assess the glycosylation status of CD147. The effects of fluvastatin, rescue compounds and inhibitors of crucial steps in the cholesterol biosynthesis pathway are shown. Alpha tubulin served as a loading control for the immunoblots. Experiments were conducted in duplicate; one representative immunoblot is shown (A). B: Quantitative analysis of LG CD147 bands shown in (A). The value of the band intensity of PMA-differentiated cells was set as 1.0. Diamonds and thirteenth column: non-glycosylated core protein (27 kDa) after tunicamycin treatment. Basal, untreated cells; PMA, PMA-differentiated cells; FS, cells treated with fluvastatin (1 μM or 10 μM). Rescue compounds: MEV, mevalonate; SQU, squalene; CHOL, cholesterol. Cholesterol pathway inhibitors: FTI-277; GGTI-298; FPT-1; AP-9, antagonistic peptide 9; TUN, tunicamycin. * on top of columns: p < 0.05, compared with PMA-differentiated cells whose value was set as 1.0. * on horizontal lines: p < 0.05, fluvastatin plus rescue compounds compared with fluvastatin only.

CD147 exists in two different glycosylated forms: a highly glycosylated, higher molecular weight (HG) form and a lowly glycosylated, lower molecular weight (LG) form. HG CD147 forms homo-oligomers and induces MMP secretion and activation, whereas LG CD147 inhibits MMP secretion. Experimentally induced changes in CD147 N-glycosylation are illustrated in Fig 6A and 6B. Treatment of THP-1 cells with fluvastatin induced LG CD147 expression in a dose-dependent manner. Rescue with mevalonate, squalene, or cholesterol was able to reduce the fluvastatin-induced increase in expression of LG CD147. Inhibition of isoprenylation by FTI-277, GGTI-298 or FPT1 (a combined inhibitor of farnesylation and geranylgeranylation) increased LG CD147. Consistently, treatment with AP-9, a specific peptide antagonist of CD147, increased LG CD147. Treatment of THP-1 cells with tunicamycin affected the levels of both LG and HG CD147: the expression of LG CD147 (33 kDa) and HG CD147 (51 kDa) was greatly diminished. The molecular weight of the form that appeared upon tunicamycin treatment (27 kDa) is consistent with that of the non-glycosylated core protein. The small amount of 51 kDa HG CD147 remaining was likely synthesized before tunicamycin treatment (Fig 6A).

### Statins impair translocation of CD147 to the cell surface

**Fig 7:**
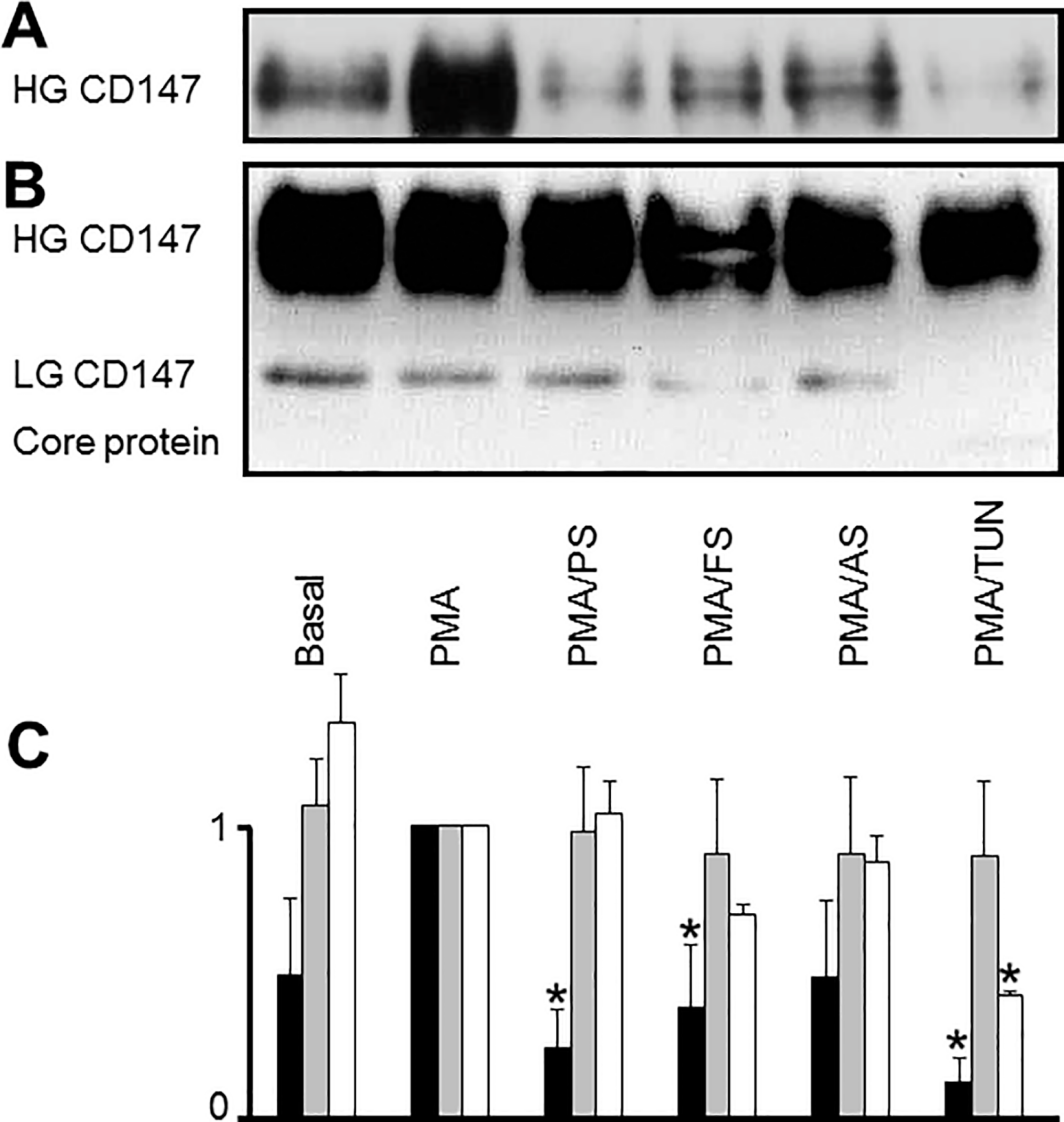
Downregulation of *de novo* synthesized CD147 glycoprotein at the cell surface after statin treatment. Levels of biotinylated cell surface proteins were compared with cellular total protein levels (in whole cell lysates) by immunoblot. A: biotinylated cell surface proteins; B: whole cell lysates. Note that the lowly glycosylated (LG) form of CD147 was not present on the cell surface and that the expression of the highly glycosylated (HG) form of CD147 was reduced predominantly on the cell surface. Experiments were conducted in duplicate; one representative immunoblot is shown. C: Quantitative analysis of bands shown in (A) and (B). The value of the band intensity of PMA-differentiated cells was set as 1.0, respectively. Black bars: HG CD147 on the cell surface. Grey bars: HG CD147 in whole cell lysate. White bars: LG CD147 in whole cell lysate. Basal, untreated cells; PMA, PMA-differentiated cells; PS, pravastatin; AS, atorvastatin; FS, fluvastatin; TUN, tunicamycin. * p < 0.05, compared with PMA-differentiated cells whose value was set as 1.0.

To confirm the results from the flow cytometry experiments comparing permeabilized and non-permeabilized THP-1 cells and to quantify intracellular retention, surface biotinylation was performed to measure the expression of CD147 on the cell surface. Cell surface levels of HG-CD147 were markedly downregulated after treatment with any of the three statins. The control substance tunicamycin decreased the translocation of CD147 to the cell surface (Fig 7A and 7C) to a greater degree than the statins. Consistently, the biotinylated (cell surface) form of the core protein could not be detected by western blot; both HG-CD147 and LG-CD147 were detected in the cell lysate (Fig 7B and 7C).

### Statins downregulate matrix metalloproteinase activity

**Fig 8:**
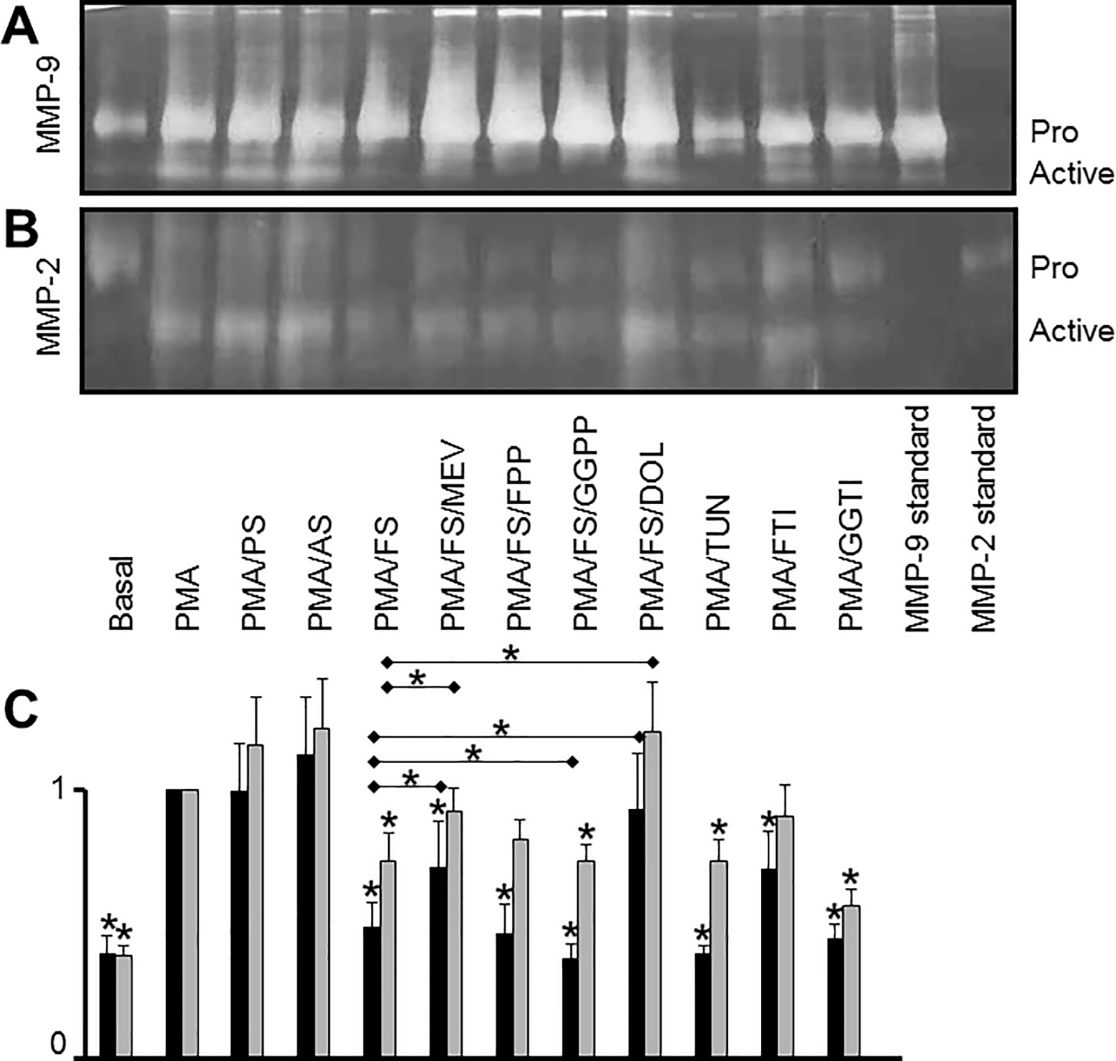
Effects of statins and controls on MMP activity. THP-1 cells were treated as described in Fig 1. Supernatants were collected and analyzed for MMP digestion activity by gelatin zymography. MMPs were identified according to their size [35] and given standards: pro-MMP-9: 92 kDa; active MMP-9: 82 kDa; pro-MMP-2: 72 kDa; active MMP-2: 62 kDa). A: zymographic gelatinase activity of MMP-9 from cell supernatants. B: zymographic gelatinase activity of MMP-2 from cell supernatants. The single blot was divided into two sections to clearly distinguish the activities of MMP-9 and MMP-2. Experiments were repeated five times; one representative result is shown. C: Quantitative analysis of bands shown in (A) and (B). The value of the band intensity of PMA-differentiated cells was set as 1.0, respectively. Black bars: active MMP-9. Grey bars: active MMP-2. Pro, pro-forms of MMPs; active, active forms of MMPs; basal, untreated cells; PMA, PMA-differentiated cells; PS; pravastatin-treated cells; AS; atorvastatin-treated cells; FS, fluvastatin-treated cells. Rescue compounds: MEV, mevalonate; FPP, farnesylpyrophosphate; GGPP, geranylgeranylpyrophosphate. Cholesterol pathway inhibitors: TUN, tunicamycin; FTI-277; GGTI-298. * on top of columns: p < 0.05, compared with PMA-differentiated cells whose value was set as 1.0. * on horizontal lines: p < 0.05, fluvastatin plus rescue compounds compared with fluvastatin only.

MMP-9 exists in several forms: a pro-form (92 kDa), an active form (82 kDa), a heterodimer (135 kDa) and a homodimer (260 kDa). Gelatin zymography is able to measure the activities of all of these forms of MMP-9. PMA-induced differentiation of THP-1 cells promoted the activation of MMP-9, as indicated by the emergence of the active form of MMP-9. Treatment with fluvastatin resulted in a decrease in the levels of active MMP-9, whereas pravastatin and atorvastatin failed to do so. To rescue the effects of fluvastatin, we added mevalonate, FPP, GGPP, or dolichol. Both mevalonate and dolichol were able to increase the levels of activated MMP-9, whereas FPP and GGPP were not. Inhibition experiments revealed that tunicamycin, FTI-277 and GGTI-298 inhibited the expression of active MMP-9 (Fig 8A and 8C).

MMP-2 exists in two forms, a pro-form (72 kDa) and an active form (62 kDa). PMA differentiation upregulated the levels of active MMP-2 in THP-1 cells. Fluvastatin reduced active MMP-2 levels, whereas pravastatin and atorvastatin did not. Dolichol and mevalonate treatment rescued active MMP-2. Tunicamycin and GGTI-298 treatment mimicked the effects of fluvastatin (Fig 8B and 8C).

## Discussion

In this study we used PMA-differentiated THP-1 cells as an *in vitro* model of monocytic differentiation [8,11,36]. We are aware that the THP-1 model is artificial and does not necessarily reflect the behavior of natural monocytes and macrophages in human diseases which limits the interpretability of the data. Another limitation of this study is that–as often seen in *in vitro* models–the concentrations of compounds are higher than *in vivo*. Concentrations of the statins used in our model exceed maximum concentrations (c max) of respective statins after oral intake of highest approved dose (80 mg per day) up to 100-fold. Another limitation of our study is its observational character.

Differentiated THP-1 cells acquire an adherent phenotype, increased surface expression of both the macrophage-specific differentiation antigen CD14 and the highly glycosylated form of CD147 and activation of MMP-9 and MMP-2.

Statins inhibit all of the elements of this macrophage differentiation process, retaining THP-1 cells in a dormant state. Possible molecular mechanisms described so far include:

1. inhibition of isoprenylation of GTP-binding proteins produced through the farnesylation and/or the geranylgeranylation pathway;
2. indirect inhibition of protein *N*-glycosylation via the farnesylation process;
3. cholesterol itself contributes to the formation of so-called lipid rafts, i.e., lipid microdomains that transport and concentrate major immunological molecules [37]. By reducing cholesterol levels, statins are able to disrupt the lipid rafts, causing subsequent dissociation of the raft-associated molecules and ultimately inhibiting their interactions [37,38];
4. statins have been observed to directly inhibit leukocyte function antigen (LFA)-1 [39], a cellular adhesion molecule that plays key roles in T-cell activation and migration [40].

To elucidate the mevalonate-dependent molecular mechanisms underlying the anti-monocytic differentiation properties of statins, two approaches were applied. First, rescue experiments were performed to reverse statin effects at several key steps of the cholesterol biosynthesis pathway; second, the effects of statins were compared to those of known inhibitors of intermediates downstream of HMG-CoA reductase.

Most of the statin effects we observed were reversed by FPP and dolichol and were mimicked by tunicamycin; in contrast, direct inhibitors of farnesylation and geranylgeranylation were not as effective as the statins, indicating that the *N*-glycosylation pathway, rather than the isoprenylation pathway, is the predominant regulator of CD147.

### Role of monocytes in inflammation

During inflammation and infection, monocytes migrate from peripheral compartments to target organs where they mature and differentiate into tissue macrophages [41]. This maturation process results in the release of several inflammatory cytokines [42] and other factors, such as MMPs [43,44].

### CD147 as a key player in monocytic differentiation

CD147 is one of the major factors reported to influence the monocytic differentiation process [45]. The pivotal role of CD147 in monocyte differentiation has been confirmed by the fact that AP-9, a specific peptide antagonist of CD147, prevents monocyte differentiation [46]. Furthermore, it has been demonstrated that AP-9 significantly inhibits the secretion and activation of MMP-2 and MMP-9 by THP-1 cells, thus emphasizing the role of CD147 in regulating MMPs [11]. CD147 interacts with adhesion markers such as CD29 (β1-integrin) and CD98 (large neutral amino acid transporter 1) [47] and participates in integrin-mediated adhesion, cellular differentiation and apoptosis [48]. Inhibition of CD147 has also been shown to impair the translocation of monocarboxylate transporter (MCT)-1 to the cell surface, disrupting the cellular lactate acid shuttle; this leads to intracellular acidification and metabolic starvation. Both of these effects lead to inhibition of monocytic differentiation [49].

### Statins as potential immunomodulators

Statins are HMG-CoA reductase inhibitors that have been shown to promote beneficial effects in autoimmune conditions [50]. Although the original function of statins was to lower plasma low density lipoprotein cholesterol, cholesterol independent effects have also been demonstrated [51]. In monocytes, statins have been shown to inhibit proinflammatory responses [52], abort the functional differentiation of monocytes and inhibit MMP secretion and activation [53]. However, the mechanisms by which they regulate these processes have not yet been completely elucidated.

### Statins and CD147

We hypothesized that inhibition of monocytic differentiation by statins is mediated through CD147 as a major player in the monocyte differentiation process [11]. In this study, statins inhibited the differentiation of THP-1 cells, which was evidenced by decreases in adherence. Statin treatment also altered the morphology of THP-1 cells, thus indicating that statins interfere with cellular differentiation.

Because of the known effects of statins on farnesylation and dolichol, we hypothesized that statins influence the molecular structure of CD147. The extracellular domain of CD147 has three putative *N*-linked glycosylation sites [18] that could potentially be inhibited by statin treatment. CD147 exists in two different glycosylation states: a HG (highly glycosylated) and a LG (lowly glycosylated) form. MMP induction is promoted by HG CD147 via homo-oligomerization, whereas LG CD147 acts as an inhibitor of MMP-2 and MMP-9, probably through its affinity for caveolin [9,19]. Thus, the glycosylation status of CD147 acts as a molecular switch for the activation of MMPs [8,54]. Our results show that fluvastatin treatment increases the expression of LG CD147, confirming the role of CD147 in mediating statin-associated MMP inhibition.

Cell permeabilization studies with statin-treated THP-1 cells revealed that the statin-induced inhibition of CD147 expression was more pronounced at the cell surface compared to the intracellular compartment. We therefore hypothesized that statins regulate the expression of CD147 through post-translational mechanisms, e.g., isoprenylation, *N*-glycosylation or intracellular retention of immature pro-forms of CD147, rather than changes at the genomic level.

From these observations, we surmised that both isoprenylation and *N*-glycosylation contribute to the expression and activity of CD147 and that both of these mevalonate-dependent pathways are inhibited by treatment with statins. One possible mechanism for statin-induced effects on *N*-linked glycosylation is through dolichol, which acts as a carbohydrate donor during the *N*-glycosylation of membrane targeted proteins; dolichol production is regulated by FPP, which is downstream of mevalonate [55].

Two distinct inhibitors of isoprenylation (FTI-277, a farnesylation inhibitor, and GGTI-298, a geranylgeranylation inhibitor) only partially inhibited CD147 surface expression and MMP activation, indicating that isoprenylation plays only a minor role in these pathways. However, the same isoprenylation inhibitors did induce the expression of LG forms of CD147. We speculate that this discrepancy is due to a potential effect of statins on caveolin-1 itself (mediated by isoprenylation) because upregulation of caveolin-1 also promotes LG CD147 forms and subsequently decreases self-association of CD147 on the cell surface [18]. Thus, statins act on isoprenylation pathways to impair trafficking of CD147 to the cell surface. Tunicamycin specifically inhibits the *N*-glycosylation of newly synthesized proteins [56]. In our experiments, tunicamycin drastically reduced *de novo N*-glycosylation of CD147, resulting in greatly increased expression of the non-glycosylated core protein. Taken together, these data suggest that statins regulate CD147 on multiple levels, particularly through isoprenylation and *N*-glycosylation.

Most transmembrane proteins are not as sensitive to changes in glycosylation status as CD147. The requirement for high levels of glycosylation for the MMP stimulating activity of CD147 [18] is unusual but not unique. Like CD147, insulin-like growth factor (IGF)-1 receptor is a membrane-targeted molecule that requires *N*-glycosylation for its proper function; reduced glycosylation activity causes proreceptor retention within the endoplasmic reticulum [32]. Consistent with our findings for CD147, two other groups have described downregulation of IGF-1 receptor by statin treatment and demonstrated that this inhibitory effect is via the isoprenylation and *N*-glycosylation pathways, which act synergistically to promote IGF-1 receptor activity [32,57].

## Conclusion

Taken together, previous reports have shown that statins

1. inhibit the upregulation of CD147 observed during monocytic differentiation [12];
2. affect transmembrane glycoproteins by inhibiting isoprenylation and dolichol-mediated *N*-glycosylation [32]; and
3. ultimately “flip” the CD147 molecular switch from an MMP inducing state to an MMP inhibiting state [9,18,19].

Further analysis of statins’ effects on other key players surrounding CD147, such as CD29, CD98, caveolin-1 and cyclophilins, will increase the understanding of the mechanisms underlying the effects of these potent pleiotropic agents.

We postulate that statins produce their anti-inflammatory effects on monocytes and macrophages by influencing CD147, making them interesting candidates for therapeutic strategies against autoimmune disorders such as MS or RA [58,59]. However, formal proof of their clinical efficacy is still pending. In contrast, statins are already widely used for primary prevention of atherosclerosis [29]. Other CD147-mediated properties of statins, such as their effects on cancer, merit further research [10,60].
